# Low-overpotential and highly sensitive detection of NADH with electrochemically pretreated cup-stacked carbon nanofiber electrodes

**DOI:** 10.1007/s44211-025-00776-y

**Published:** 2025-05-02

**Authors:** Sota Goto, Taiyo Iwasaki, Kikuo Komori

**Affiliations:** 1 Graduate School of System Engineering, Kindai University, Takaya-Umenobe, Higashi-Hiroshima, 739-2116 Japan; 2 Department of Biochemistry and Chemistry, Kindai University, Takaya-Umenobe, Higashi-Hiroshima, 739-2116 Japan

**Keywords:** β–Nicotinamide adenine dinucleotide, Graphene edges, Electrochemical pre-reduction treatment, Low-overpotential, Biosensing

## Abstract

Cup-stacked carbon nanofibers (CSCNFs), the surface of which provides highly ordered graphene edges and electroactive oxygen-containing functional groups, were investigated as electrode materials for oxidation of β–Nicotinamide adenine dinucleotide (NADH) at low overpotential. The NADH oxidation was facilitated at the CSCNF-modified glassy carbon (CSCNF/GC) electrode, the surface of which was electrochemically activated at –0.40 V (vs. Ag|AgCl) to generate electrochemically reduced electroactive oxygen-containing functional groups. The anodic peak current of NADH oxidation was observed at about + 0.065 V by cyclic voltammetry measurements. The obtained value was negatively shifted by ~ 0.33 and ~ 0.63 V compared with the CSCNF/GC electrode without the electrochemical reductive pretreatment and the GC electrode, respectively. In addition, NADH-dependent glucose dehydrogenase (GDH) molecule-modified CSCNF/GC electrodes enabled the detection of glucose at the low overpotential effectively in the presence of NAD^+^, which accepts electrons from GDH and then reduces to NADH, after the electrochemical reductive pretreatment. Thus, the electrochemical reductive pretreatment of CSCNF electrodes would be useful for the development of highly sensitive NADH-dependent enzymatic biosensors.

**Graphical abstract:**

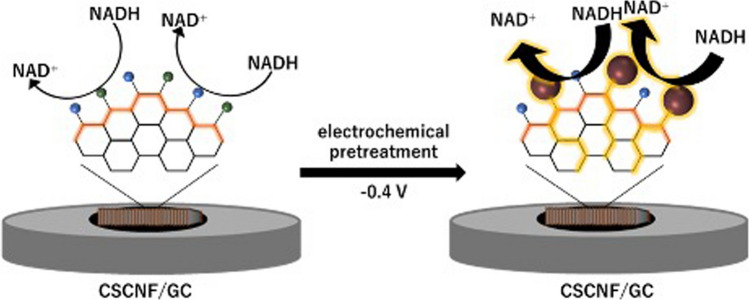

## Introduction

β–Nicotinamide adenine dinucleotide (NADH) known as a cofactor for more than 300 dehydrogenase enzymes has widely been used for an index in biosensing applications. In the electrochemistry field, the direct electrochemical oxidation of NADH is of great interest to play the role of electron and hydrogen shuttle between electrodes and the enzymes reduced by substrates [1, 2]. However, it has been known that the overpotential is required for the electrocatalytic oxidation of NADH. For instance, NADH is usually oxidized at + 1.1 and + 1.3 V (vs. Ag|AgCl) or larger at carbon [3] and platinum [4] electrodes, respectively. At such a potential range, as not only NADH but also other interferences in actual samples should be oxidized, it is difficult to determine the exact anodic current responses of only NADH oxidation. Hence, a decrease in the overpotential for the electrochemical oxidation of NADH is one of the significant challenges.

To reduce the large overpotential for the direct electrochemical oxidation of NADH, various types of electrodes have been developed and investigated by many electrochemists. Quinone derivatives adsorbed at the glassy carbon (GC) electrode surface are known to electrochemically catalyze NADH oxidation efficiently [5–9]. In particular, *o*-quinones exhibit less stable redox-active molecules but are effective in the electrocatalytic oxidation of NADH, compared with *p*-quinones [6]. An anodic peak for the NADH oxidation was known to be observed at about + 0.15 and + 0.185 V for graphite electrodes modified with 4-[2-(1-pyrenyl)vinyl]catechol [10] and 4-[2-(2-naphthyl)vinyl]catechol [11], respectively, by cyclic voltammetry measurements. Compared with the bare graphite electrode (ca. + 0.41 V), those peak potentials were shifted to more negative values. Additionally, it has been reported that the anodic peak at GC electrodes modified with acid-treated single-walled and multi-walled carbon nanotubes (SWCNTs and MWCNTs) were shifted to + 0.46 and + 0.49 V, respectively, compared with that at the bare GC electrode (ca. + 0.82 V), probably due to redox mediation of NADH oxidation through the oxygen-containing functional groups, such as quinone groups [12]. It is also well-known that for crystalline graphite, edge plane sites are electroactive, whereas the basal plane is less active [13, 14]. Actually, the peak potential of adenine oxidation at an edge plane pyrolytic graphite electrode (ca. + 0.95 V) was reported to be shifted to a negative value, compared with that at a basal plane pyrolytic graphite electrode (ca. + 1.07 V) [15], demonstrating that the electrochemical oxidation of NADH was facilitated at the edge plane sites. Additionally, the negative peak potential shift by ~ 0.57 V was also observed at the GC electrode modified with acid-treated carbon nanofibers (CNFs), which provide a high density of electroactive exposed edge plane sites with oxygen-containing functional groups, compared with the case at the GC electrode [16]. Besides them, an electrochemically pre-anodized screen-printed carbon electrode, which exhibits the surface comprising electroactive edge plane sites with oxygen-containing functional groups through the pre-anodization, also enabled the decrease in the overpotential of NADH oxidation (ca. + 0.4 V) [17]. From these previous reports, the edge plane sites with oxygen-containing functional groups including quinone groups might play an important role in the NADH oxidation at much lower overpotentials, but the details are still completely unclear.

In recent years, we have used cup-stacked carbon nanofibers (CSCNFs) [18–21] and graphite nanofibers (GNFs) [22] as electrode materials. These carbon nanomaterials provide highly ordered graphene edges, compared with SWCNTs consisting of graphene cylinder with open ends, allowing for fast electrochemical communication with redox species including redox enzymes [23, 24]. We have also reported that the oxygen- and nitrogen-containing functional groups at the CSCNF surface contributed to the electron transfer kinetics of redox species, such as [Fe(CN)_6_]^3–/4–^ and Fe^2+/3+^ [25, 26]. For instance, the electron transfer rate of the former was decelerated and that of the latter was accelerated by the oxygen-containing functional groups, mainly due to the electrostatic repulsion and attraction, respectively [25]. In addition, the surface functional groups at the CSCNF electrode enabled to regulate the orientation of enzymes, such as fructose dehydrogenase, to improve the direct electrochemical communication with enzymes [27]. As the geometric surface structure of CSCNFs is broadly analogous to that of stacked carbon nanofibers with highly ordered graphene edges, the CSCNF electrode should be useful in understanding the mechanism of NADH oxidation. In the present work, we examined electrochemical responses to the NADH oxidation at CSCNF-modified GC (CSCNF/GC) electrodes. We found that electrochemically reduced electroactive oxygen-containing functional groups at the surface of the CSCNF/GC electrode was involved in facilitating the NADH oxidation remarkably at the low overpotential. We further demonstrated effective detection of glucose using the CSCNF/GC electrode modified with glucose dehydrogenase (GDH) as the typical NADH-dependent dehydrogenase in the presence of NAD^+^, which accepts electrons from GDH and then reduces to NADH, after the electrochemical reductive pretreatment. 

## Experimental

CSCNFs (Vision Development Co. Ltd., Japan) were dispersed in an aqueous solution containing 30% HNO_3_ for 120 min at 90 ˚C to oxidize their surfaces. The oxidized CSCNFs were also heated at 300, 400, and 500 ˚C for 120 min under an argon atmosphere to give partially deoxygenated CSCNFs. We hereby prepared the other three types of CSCNFs, such as 300-, 400-, and 500-CSCNFs. Preparation methods of the GC electrode modified with CSCNFs are as follows. The GC electrode (3 mm in diameter, ALS Co., Ltd.) was polished with 0.3 and 0.05 µm alumina slurries on a polishing cloth and then sonicated in methanol and acetone each for 5 min. After drying, an aliquot of 1.0 mg mL^–1^ CSCNF dispersion in *N*,*N*-dimethylformamide (DMF) was cast onto the GC surface (ca. 120 µg cm^–2^), followed by drying at room temperature. The obtained CSCNF/GC electrode was further dried in an electric oven at 40 ˚C. In addition, for GC electrodes modified with any of 300-, 400-, and 500-CSCNFs (300-, 400-, and 500-CSCNF/GC electrodes), we used the same protocol for the CSCNF/GC electrode. X-ray photoelectron spectroscopy (XPS, PHI5000, ULVAC-PHI, Inc.) and Raman spectroscopy (NRS-3300, JASCO Corp.) were used for the characterization of the CSCNF surface.

Fundamental electrochemical properties of CSCNF/GC electrodes were evaluated by cyclic voltammetry and amperometry in a 67 mM phosphate buffer (pH 7.4) containing NADH (FUJIFILM Wako Pure Chem. Co., Japan) using a potentiostat SP-150 (Bio-Logic Science Instruments, Ltd.). A Ag|AgCl|KCl (sat.) and a coiled platinum wire were used as reference and counter electrodes, respectively. Current densities normalized by the geometrical surface area of the GC electrode were used in electrochemical measurements.

For glucose sensing, we prepared CSCNF/GC electrodes modified with NADH-dependent GDH (TOYOBO Co., Ltd., Japan), as follows. The 67 mM phosphate buffer (pH 7.4) containing 6.9 µM GDH was cast onto the surface of the CSCNF/GC electrode, followed by drying in the refrigerator (ca. 4 ˚C). Subsequently, 0.2% Nafion solution was coated to retain the GDH molecules onto the CSCNF/GC electrode surface. After thoroughly rinsing with distilled water, we obtained GDH-modified CSCNF/GC (GDH/CSCNF/GC) electrodes. The obtained working electrode was immersed in the 67 mM phosphate buffer containing 10 mM NAD^+^ and 0–30 mM glucose. The working electrode was pre-polarized at –0.4 V (vs. Ag|AgCl) for 10 s and then switched to 0 V. From the obtained current, electrochemical properties of the GDH/CSCNF/GC electrode to glucose were evaluated.

## Results and discussion

**Fig. 1:**
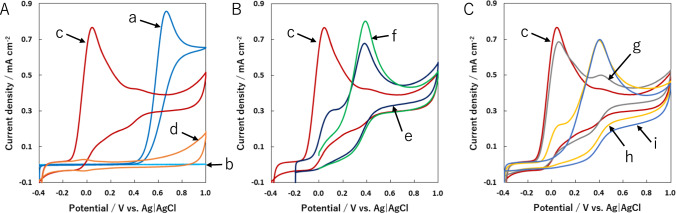
**A** Cyclic voltammograms for GC (**a** and **b**) and CSCNF/GC (**c** and **d**) electrodes in 67 mM phosphate buffer (pH7.4) with (**a** and **c**) and without (**b** and **d**) 10 mM NADH. **B** Comparison of cyclic voltammograms for CSCNF/GC electrodes at the initial potential of (**c**) –0.4, (**e**) –0.2, and (**f**) 0.0 V in 67 mM phosphate buffer (pH 7.4) containing 10 mM NADH. **C** Comparison of cyclic voltammograms for (**c**) CSCNF/GC (without thermal treatment), (**g**) 300-CSCNF/GC, (**h**) 400-CSCNF/GC, and (i) 500-CSCNF/GC electrodes in 67 mM phosphate buffer (pH7.4) containing 10 mM NADH. Scan rate was 10 mV s^–1^

We first examined the electrochemical behavior of NADH at GC and CSCNF/GC electrodes in the phosphate buffer (pH 7.4). Figure 1 shows cyclic voltammograms (CVs) at a scan rate of 10 mV s^–1^. As the potential was swept from –0.40 to + 1.0 V, an anodic current with a peak potential of + 0.70 V was observed at the GC electrode (Fig. 1A, curve a). This value agreed well with the previous report [12]. In contrast, at the CSCNF/GC electrode with oxygen-containing functional groups, the anodic peak potential shifted to lower potentials (ca. + 0.065 V) in comparison with the case for the GC electrode (Fig. 1A, curve c). Note that no Faradaic oxidation current was observed at the CSCNF/GC electrodes between –0.4 and + 1.0 V in the phosphate buffer containing NAD^+^ (Figure S1). The anodic current with the peak potential of + 0.065 V was therefore based on the electrochemical oxidation of the nicotinamide moiety in NADH. The peak potential value obtained here was nearly equal to that at oxidized carbon nanofiber-modified GC electrodes reported previously [16]. Meanwhile, as the potential was swept from –0.20 to + 1.0 V, the anodic current decreased at + 0.065 V and increased at about + 0.40 V (Fig. 1B, curve e). In the potential range between 0 and + 1.0 V, the anodic current further increased at about + 0.40 V (Fig. 1B, curve f). These differences might be derived from the amount of reduced carboxyl and carboxylic anhydride groups on the CSCNF surface. It has been known that the carboxyl and carboxylic anhydride groups on the surface of a carbon material is desorbed by thermal treatment at 400 ˚C or higher under an inert gas [18, 28]. We therefore examined the electrochemical behavior of NADH between –0.4 and + 1.0 V at the GC electrode modified with CSCNFs after the thermal treatment (Fig. 1C). For the 300-CSCNF/GC electrode, the anodic current based on NADH oxidation slightly decreased at + 0.065 V and increased at about + 0.40 V (Fig. 1C, curve g), compared with the case for the CSCNF/GC electrode (Fig. 1C, curve c). However, for the 400-CSCNF/GC electrode, the anodic current significantly decreased and increased at the former and latter potentials, respectively (Fig. 1C, curve h). In addition, for the 500-CSCNF/GC electrode, the anodic current with one peak potential at + 0.40 V was observed (Fig. 1C, curve i). This value was about 0.30 V smaller than that for the GC electrode (+ 0.70 V) presumably thanks to the effect of electroactive graphene edges at the 500-CSCNF surface. The peak potential value obtained here also agreed well with that of the GC electrode modified with stacked graphene platelet nanofibers, which provide highly ordered graphene edge sites, as reported previously [29]. Thus, electroactive oxygen-containing functional groups and graphene edge sites at the CSCNF surface might strongly contribute to the indirect (electrocatalytic) and direct oxidation reactions of NADH at + 0.065 and + 0.40 V, respectively.

**Fig. 2:**
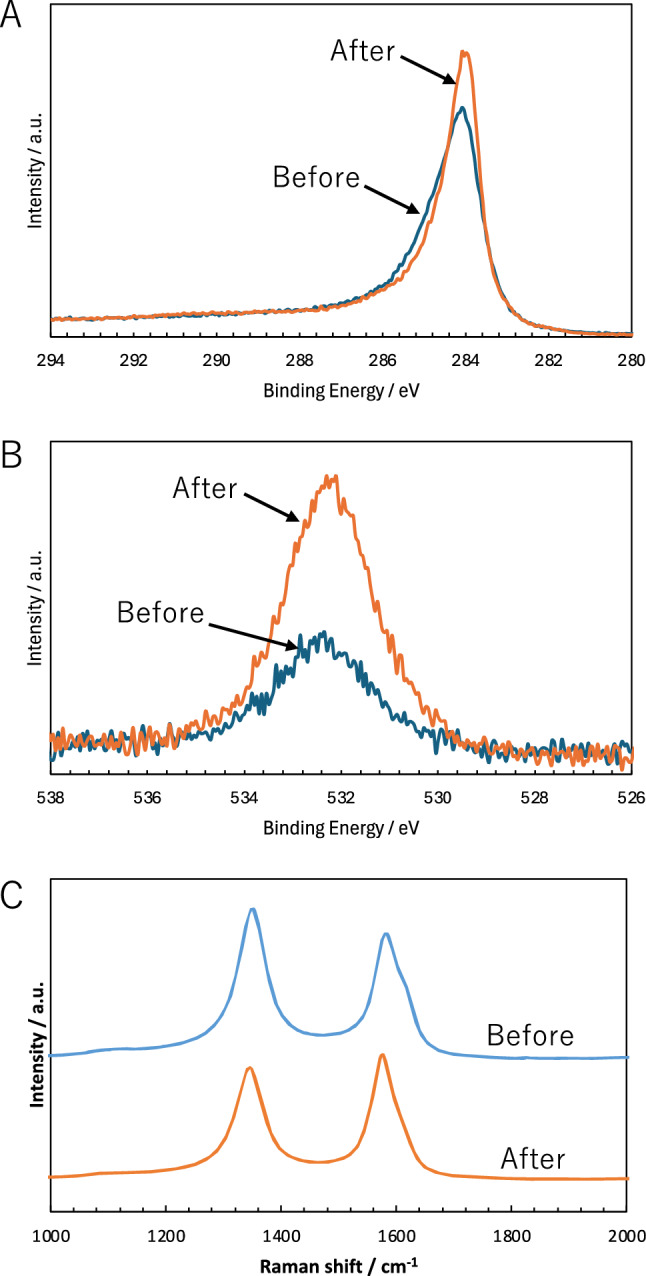
**A** C1s and **B** O1s XPS spectra and **C** Raman spectra of the CSCNF electrode surface before and after applying potential of –0.40 V for 10 s in 67 mM phosphate buffer (pH 7.4)

Based on the above results, we discussed as follows. Electroactive oxygen-containing functional groups on the CSCNF surface likely exhibited good electrocatalytic activity for the oxidation of NADH. Indeed, a reversible redox peak likely based on electroactive carboxyl and carboxylic anhydride groups was observed at about –0.011 V for the CSCNF/GC electrode without thermal treatment, but not for 400-CSCNF/GC and 500-CSCNF/GC electrodes with thermal treatment (Figure S2) [30, 31]. As mentioned above, it must be emphasized that the significant anodic current with the peak potential of + 0.065 mV was observed when the potential sweep started at the initial potential of –0.40 V, but not that of –0.20 V or larger. In this context, the reduced carboxyl and carboxylic anhydride groups were implicated to accelerate the electrochemical oxidation of NADH with the CSCNF/GC electrode. In response to this result, we measured XPS for the CSCNF surface before and after applying a potential of –0.40 V for 10 s in 67 mM phosphate buffer (pH 7.4) (Fig. 2A and B). The peak intensity of C1s spectrum corresponding to predominantly C–C moiety [32] increased after the applying potential. To confirm this, we also observed Raman spectra (Fig. 2C). It has been known that typical sp^2^ carbon atoms give a *G* band signal at ~ 1580 cm^–2^, whereas carbon atoms adjacent to a defect or a graphene edge also give a *D* band signal at ~ 1350 cm^–2^ [33, 34]. The relative intensity ratio of the *D* and *G* band peaks (*I*_D_/*I*_G_ ratio) is often employed for the characterization of sp^2^ carbon nanomaterials to estimate defects in their graphite structure [35]. The *I*_D_/*I*_G_ ratios were determined to be about 1.20 and 0.89 before and after applying the potential of –0.40 V, respectively. In addition, the graphene edge also gives a *D*’ band signal at ~ 1620 cm^–2^ [36]. A shoulder based on the *D*’ band signal after applying the potential of –0.40 V was smaller than that for no applying potential. These results might be supportive of the above XPS result. Meanwhile, the peak intensity of O1s spectrum corresponding to C = O, O–C = O, COOH, C–OH moieties [32, 37, 38] for the CSCNF surface after applying the potential was larger than that before applying the potential (Fig. 2B). Concurrently, we compared the peak binding energy of O1s spectrum for the CSCNF electrode surface with that for the 500-CSCNF electrode surface, but almost no difference was observed. Therefore, carboxyl and carboxylic anhydride groups might not be completely desorbed from the 500-CSCNF surface after the thermal treatment. However, based on the results in Fig. 1, the electroactive oxygen-containing functional groups including carboxyl and carboxylic anhydride groups would play an important role to accelerate electrochemical oxidation of NADH at the CSCNF surface. Unfortunately, as a specific reduced form of electroactive oxygen-containing functional groups was not determined in the present study, further study is needed. On the other hand, the anodic current with a peak potential of + 0.065 V decreased upon repeated scans in cyclic voltammetry measurements during –0.40 and + 1.0 V. The most plausible reason relates to an electrode fouling due to the coupling reaction between reactive intermediate radicals through the electrochemical oxidation process of NADH and the reduced carboxyl groups on the CSCNF surface, resulting in the blocking of effective reaction sites [10]. Therefore, present CSCNF-modified electrodes for the detection of NADH hold promise for use as disposable electrochemical sensors.

**Fig. 3:**
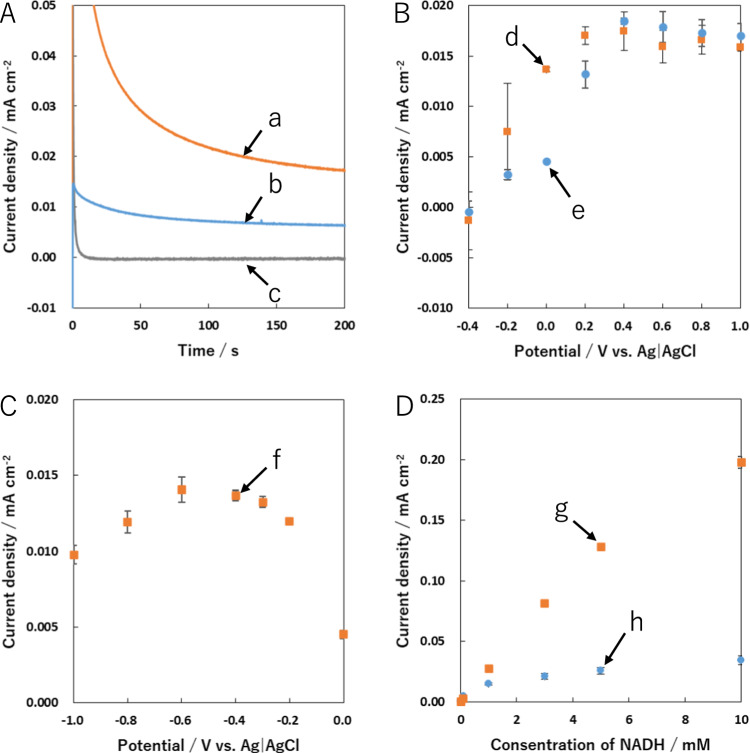
**A** Changes in anodic current densities at 0.0 V for the CSCNF electrode with (**a** and **c**) and without (**b**) electrochemical pretreatment at –0.40 V for 10 s in 67 mM phosphate buffer (pH 7.4) containing 1.0 mM NADH (**a** and **b**) or no NADH (**c**). **B** Dependences of anodic current densities collected at 200 s on the applied potential to the CSCNF electrode with (**d**) and without (**e**) the pretreatment. **C** Correlation between anodic current densities collected at 200 s after switching applied potential to 0.0 V and pretreatment potentials for 10 s. **D** Dependences of anodic current densities collected at 50 s on NADH concentration for CSCNF/GC electrode with (**g**) and without (**h**) the pretreatment at –0.40 V for 10 s. The plots for d-h represent the mean ± standard error of three independent electrodes

Next, amperometry measurements were performed. As described above, the reduced oxygen-containing functional groups including carboxyl and carboxylic anhydride groups on the CSCNF surface might play an important role to detect NADH at the low overpotential. We therefore measured the anodic current of NADH oxidation at the applied potential of 0.0 V after the electrochemical reductive pretreatment of the CSCNF/GC electrode at –0.40 V for 10 s (Fig. 3A, curve a). Even in the amperometry measurement, the anodic current was clearly larger than that without the pretreatment (Fig. 3A, curve b), indicating that the electrochemical reductive pretreatment contributed to the facilitation of NADH oxidation. We also examined the correlation between applied potentials after the pretreatment at –0.40 V for 10 s and anodic currents collected at 200 s after switching applied potentials (Fig. 3B). Anodic currents of NADH oxidation increased with an increase in the applied potentials up to + 0.40 V and then nearly leveled off at + 0.60 V or larger (Fig. 3B, plots d). Additionally, the anodic currents at the potential step between –0.20 V and + 0.20 V were larger than those for the CSCNF/GC electrode without the pretreatment (Fig. 3B, plots e). In particular, NADH was efficiently oxidized at the potential step of 0.0 V after the electrochemical pretreatment at –0.40 V for 10 s. Note that the observed anodic current was nearly equal to that after the electrochemical pretreatment at –0.40 V for 30 s. Thus, electroactive oxygen-containing functional groups on the CSCNF surface might be completely reduced at –0.40 V even for 10 s.

We further examined the optimal potential value of the electrochemical reductive pretreatment. The CSCNF/GC electrode was electrochemically activated by applying a constant potential of –1.0 ~ 0.0 V for 10 s and then switched to the applied potential of 0.0 V to observe the anodic current of NADH oxidation at low overpotential. Figure 3C shows the correlation between pretreatment potentials and anodic currents observed at 200 s after switching potential to 0.0 V. The maximum anodic currents were observed between –0.60 and –0.40 V. In contrast, the anodic currents gradually decreased at pretreatment potentials of –0.30 V or larger presumably due to the reduction of the electroactive oxygen-containing functional groups on the CSCNF surface insufficiently. However, even for the pretreatment potential of –0.80 V or smaller, the anodic currents decreased. This reason is unclear. The most likely reason is that reductive deoxygenation of electroactive oxygen-containing functional groups at the CSCNF surface took place by applying extremely negative potentials to the CSCNF/GC electrode, leading to its deactivation. Indeed, the steady-state cathodic currents increased as the pretreatment potential decreased down to –0.40 V and then nearly leveled off at the applied potential of –0.60 V. However, the steady-state cathodic currents further increased as the pretreatment potential further decreased down to –1.0 V, compared with those at –0.40 and –0.60 V. Collectively, electrochemical oxidation of the nicotinamide moiety in NADH was efficiently facilitated by applying the potential of 0.0 V to the CSCNF/GC electrode after the electrochemical pretreatment at –0.40 V for 10 s. Based on the present amperometry technique with the electrochemical pretreatment process, we also examined the relationship between anodic currents collected at 50 s after switching the applied potential to 0.0 V and the NADH concentration (Fig. 3D). The anodic current for the CSCNF/GC electrode with the pretreatment linearly increased with increasing NADH concentration up to 3.0 mM (Fig. 3D, plots g). The sensitivity was determined to be about 2.7 × 10^–2^ A cm^–2^ M^–1^, whereas that without the pretreatment was not determined due to no linear relationship between the anodic current densities and NADH concentration (Fig. 3D, plots h).

**Fig. 4:**
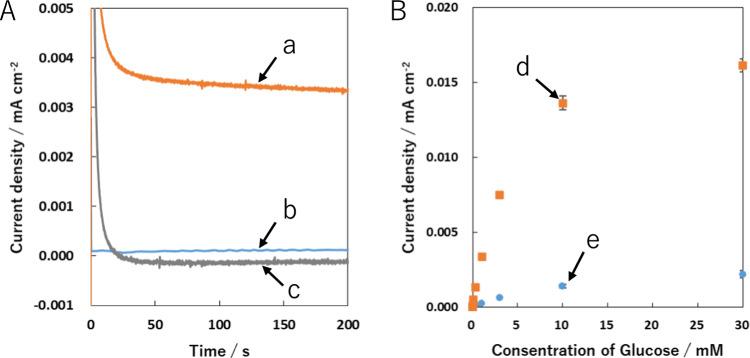
**A** Chronoamperometric responses at 0.0 V for GDH/CSCNF/GC electrodes with (**a** and **c**) and without (**b**) the electrochemical pretreatment at –0.40 V for 10 s in the air-saturated 67 mM phosphate buffer (pH 7.4) containing either 1.0 mM glucose and 10 mM NAD^+^ (**a** and **b**) or no glucose and 10 mM NAD^+^ (**c**). **B** Dependences of anodic current densities collected at 50 s on glucose concentration in the air-saturated 67 mM phosphate buffer (pH 7.4) containing 10 mM NAD^+^ for GDH/CSCNF/GC electrodes with (**d**) and without (**e**) the pretreatment. The plots for d and e represent the mean ± standard error of three independent electrodes

We further carried out a feasibility study of the CSCNF/GC electrode modified with NADH-dependent enzymes toward the development of electrochemical biodevices including biosensors and enzymatic biofuel cells. As a typical example of NADH-dependent enzymes, we used GDH, which catalyzes the oxidation of glucose via gluconolactone to gluconic acid accompanying the reduction of cofactor NAD^+^ to NADH. We performed amperometry measurements in the 67 mM phosphate buffer containing NAD^+^ and glucose. As expected, a steady-state current for the GDH/CSCNF/GC electrode after the electrochemical pretreatment clearly increased, compared with that without the pretreatment (Fig. 4A). We also found that the anodic current was hardly observed in the phosphate buffer containing either NAD^+^ or glucose. The anodic current observed here is therefore based on the electron transfer from NADH, which is generated from glucose oxidation catalyzed by GDH to the electrochemically pre-reduced CSCNF/GC electrode surface. The possible reaction mechanisms are as follows.

1 $$Glucose+{NAD}^{+}\stackrel{GDH}{\to }Gluconolactone+NADH+{H}^{+}$$

2 $$NADH\to {NAD}^{+}+{H}^{+}+2{e}^{-}$$

Figure 4B shows the dependences of anodic currents collected at 50 s after switching the applied potential to 0.0 V on the glucose concentration in the phosphate buffer (pH 7.4) containing 10 mM NAD^+^. The anodic currents for the GDH/CSCNF/GC electrode with the pretreatment increased up to 300 µM glucose, indicating that reaction 1 was the rate-determining step of the reaction (Fig. 4B, plots d). The sensitivity in the linear range of 1–300 µM was determined to be about 4.6 × 10^–3^ A cm^–2^ M^–1^. This value was about 23 times larger than that for the GDH/CSCNF/GC electrode without the pretreatment (ca. 2.0 × 10^–4^ A cm^–2^ M^–1^, Fig. 4B, plots e). The plateau of the anodic current (*I*_max_) at the glucose concentration above 10 mM was almost independent of the glucose concentrations, likely indicating that reaction 2 is the rate-determining step. The anodic currents observed here were also found to be kinetically controlled but not diffusion controlled, because the current response didn’t increase even if the electrolyte solution was stirred. According to these results, the obtained electrode exhibits characteristics of the Michaelis–Menten kinetic mechanism. The steady-state current *I* based on the glucose oxidation could therefore be expressed as the following Lineweaver–Burk type equation [39]:

3 $$\frac{1}{I}=\frac{{K}_{m}^{app}}{{I}_{max}}\left(\frac{1}{[C]}\right)+\frac{1}{{I}_{max}}$$

where ${K}_{m}^{app}$ is the apparent Michaelis constant and *C* is the glucose concentration. Based on the pseudo-Lineweaver–Burk plot (1/*I* vs. 1/[*C*], Figure S3), *I*_max_ and ${K}_{m}^{app}$ values for the GDH/CSCNF/GC electrode with the pretreatment were determined to be about 1.6 × 10^–5^ A cm^–2^ and 3.2 mM, respectively. The former value was about 10 times larger than that for the GDH/CSCNF/GC electrode without the pretreatment (ca. 1.6 × 10^–6^ A cm^–2^). This result clearly indicates that the rate of electron transfer from NADH to the CSCNF surface after the electrochemical pretreatment was accelerated, as described above. Nevertheless, the obtained ${K}_{m}^{app}$ value was slightly smaller than that without the pretreatment (ca. 4.2 mM). In Fig. 4B, the slope in the linear range should be involved in the reaction rate of Eq. 1. As mentioned above, the sensitivity for the GDH/CSCNF/GC electrode with the pretreatment was larger than that without the pretreatment. This difference is likely related to the degree of structural distortion of GDH molecules adsorbed at the CSCNF/GC electrode surface. Intriguingly, the electrochemical pretreatment might mitigate the degree of the structural distortion based on changes in electrostatic interaction and repulsion between the enzyme and CSCNF surface, leading to the acceleration of the reaction rate for Eq. 1. However, the details are still unclear. Incidentally, the obtained ${K}_{m}^{app}$ value for the GDH/CSCNF/GC electrode with the pretreatment was smaller than that of the free enzyme in solution (13.8 mM) [40]. Enzymes attached to the electrode surface frequently cause structural distortion and retardation of catalytic reaction rates, leading to low values of *K*_m_. Therefore, the obtained values might be reasonable. On balance, the GDH/CSCNF/GC electrode after the electrochemical pretreatment was beneficial for the low-overpotential and highly sensitive determination of glucose in the presence of NAD^+^, compared with that without the pretreatment. We believe that even if other NADH-dependent enzymes, such as alcohol dehydrogenase and glycerol dehydrogenase, are employed instead of GDH, specific substrates for each enzyme should be detected. To elucidate this, further studies are needed.

## Conclusion

We successfully observed anodic peak current responses for NADH oxidation at low overpotential (ca. + 0.065 V) using the CSCNF/GC electrode after the electrochemical reductive pretreatment. In addition, the CSCNF/GC electrode modified with NADH-dependent GDH after the electrochemical pretreatment at –0.40 V for 10 s was applicable to the effective detection of glucose in the presence of NAD^+^. The sensitivity in the linear range of 1–300 µM glucose for the GDH/CSCNF/GC electrode with the pretreatment was found to be about 23 times larger than that without the pretreatment. Unfortunately, oxidation products of NADH caused the surface fouling of the CSCNF/GC electrode. However, since dispersed CSCNFs allow easy coating and patterning of their films for electrodes, the present CSCNF electrode surface activated by the electrochemical reductive pretreatment process might be applied to disposable NADH-dependent enzymatic-based electrochemical biosensors.

## Data Availability

Data will be made available on request.
